# Intralaminar and medial thalamic influence on cortical synchrony, information transmission and cognition

**DOI:** 10.3389/fnsys.2014.00083

**Published:** 2014-05-09

**Authors:** Yuri B. Saalmann

**Affiliations:** Department of Psychology, University of Wisconsin—MadisonMadison, WI, USA

**Keywords:** neural synchrony, memory, attention, reward, schizophrenia, anesthesia, reuniens nucleus, mediodorsal nucleus

## Abstract

The intralaminar and medial thalamic nuclei are part of the higher-order thalamus, which receives little sensory input, and instead forms extensive cortico-thalamo-cortical pathways. The large mediodorsal thalamic nucleus predominantly connects with the prefrontal cortex, the adjacent intralaminar nuclei connect with fronto-parietal cortex, and the midline thalamic nuclei connect with medial prefrontal cortex and medial temporal lobe. Taking into account this connectivity pattern, it is not surprising that the intralaminar and medial thalamus has been implicated in a variety of cognitive functions, including memory processing, attention and orienting, as well as reward-based behavior. This review addresses how the intralaminar and medial thalamus may regulate information transmission in cortical circuits. A key neural mechanism may involve intralaminar and medial thalamic neurons modulating the degree of synchrony between different groups of cortical neurons according to behavioral demands. Such a thalamic-mediated synchronization mechanism may give rise to large-scale integration of information across multiple cortical circuits, consequently influencing the level of arousal and consciousness. Overall, the growing evidence supports a general role for the higher-order thalamus in the control of cortical information transmission and cognitive processing.

## Introduction

**Figure 1:**
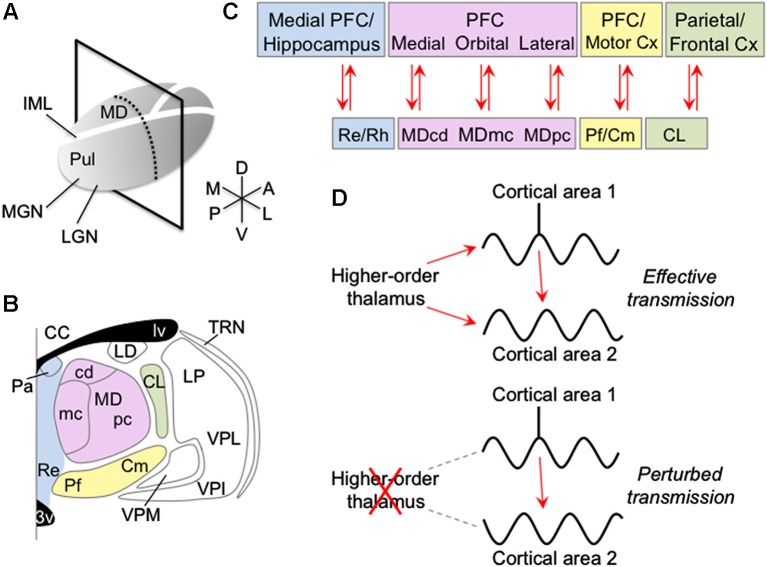
**Anatomy of the intralaminar and medial thalamic nuclei and their connectivity with the cortex. (A)** Right thalamus overview, showing plane of section in **panel B**. **(B)** Coronal view of thalamus showing anterior intralaminar (green), posterior intralaminar (yellow), MD (pink) and midline (blue) nuclei. **(C)** Cortical connections of intralaminar and medial nuclei. **(D)** Schematic showing proposed mechanism of how the higher-order thalamus influences the cortex. The higher-order thalamus adjusts the magnitude and phase of synchrony between different groups of cortical neurons. Synchronizing cortical neurons, such that action potentials from pre-synaptic neurons arrive during phases of increased excitability of post-synaptic neurons, can increase the efficacy of their information exchange (top). In contrast, abnormal higher-order thalamic function can perturb cortico-cortical information transmission, either by reducing transmission efficacy with possible information degradation (bottom), or by erroneously routing information across cortex. Abbreviations: 3v, third ventricle; CC, corpus callosum; CL, central lateral nucleus; Cm, centromedian nucleus; Cx, cortex; IML, internal medullary lamina; LD, lateral dorsal nucleus; LGN, lateral geniculate nucleus; lv, lateral ventricle; MD, mediodorsal nucleus; MDcd, caudodorsal division of MD; MDmc, magnocellular division of MD; MDpc, parvocellular division of MD; MGN, medial geniculate nucleus; Pa, paraventricular nucleus; Pf, parafascicular nucleus; PFC, prefrontal cortex; Pul, pulvinar; Re, reuniens nucleus; Rh, rhomboid nucleus; TRN, thalamic reticular nucleus; VPI, ventral posterior inferior; VPL, ventral posterior lateral nucleus; VPM, ventral posterior medial nucleus.

Information from the sensory periphery is first transmitted to the cerebral cortex via the primary sensory, or first-order, thalamic nuclei, such as the lateral geniculate in visual thalamus, ventral division of the medial geniculate in auditory thalamus, and ventral posterior nuclei in somatosensory thalamus. These first-order thalamic nuclei also receive feedback from the cortex, from layer 6. In contrast, higher-order thalamic nuclei, such as the pulvinar, mediodorsal (MD), intralaminar and midline nuclei (Figure 1), receive relatively little input from the sensory periphery. Instead, these higher-order thalamic nuclei receive major input from cortical layer 5 as well as cortical layer 6, and project to the cerebral cortex to form prevalent cortico-thalamo-cortical pathways (Guillery, 1995; Sherman and Guillery, 2002). This provides indirect connections between cortical areas via the higher-order thalamus, in addition to the direct cortico-cortical connections (Shipp, 2003; Sherman and Guillery, 2006). Although the direct cortico-cortical connections are commonly thought to convey detailed perceptual and cognitive information between cortical areas (but see Sherman and Guillery, 2006), the function of the higher-order thalamus and its connections with the cortex are poorly understood.

Lesions of higher-order thalamic nuclei have been shown to impair a number of cognitive functions, including memory, attention, perception and sensory-guided actions (Mitchell et al., 2008; Snow et al., 2009; Wilke et al., 2010). Although the underlying mechanism is unclear, recent evidence suggests that higher-order thalamic lesions perturb cortico-cortical information transmission (Theyel et al., 2010; Purushothaman et al., 2012). One possible mechanism may involve the higher-order thalamus modulating the degree of synchrony between different cortical neurons (Saalmann et al., 2012). Synchronizing cortical neurons can increase efficacy of information transmission. Synchronized pre-synaptic neurons are more likely to drive the post-synaptic neuron. Further, synchronized oscillatory activity of pre- and post-synaptic neurons, such that spikes from a pre-synaptic neuron arrive during periods of reduced inhibition of a post-synaptic neuron, increases the likelihood of spikes being relayed (Aertsen et al., 1989; Fries et al., 2001; Saalmann et al., 2007; Womelsdorf et al., 2007; Gregoriou et al., 2009; Tiesinga and Sejnowski, 2009). In this scenario, the higher-order thalamus may synchronize one network of cortical neurons and desynchronize other cortical networks, thereby selectively transmitting behaviorally relevant information between appropriately synchronized cortical neurons. This mechanism has the advantage of being able to dynamically route information across the cortex according to behavioral demands, by synchronizing different networks of neurons at different times. This review discusses how the relatively little-explored intralaminar and medial thalamic nuclei may influence the cortex. Although these higher-order thalamic areas, and even their subdivisions (Figures 1B, C), may contribute to different cognitive functions, they show similar thalamo-cortical connectivity principles. Here, I argue that this connectivity gives rise to common thalamic-mediated mechanisms for regulating cortical oscillation and synchronization patterns.

## Anatomy of the intralaminar and medial thalamus

The internal medullary lamina contains myelinated fibers that course through the thalamus along its rostro-caudal axis (Figure 1A; this review focuses on thalamo-cortical networks in primates; references to cat and rodent data are noted below). The large MD nucleus, and adjacent midline structures, the paratenial, paraventricular, reuniens (Re) and rhomboid (Rh) nuclei, are located medial to the internal medullary lamina. Within the lamina, there are also several nuclei. These intralaminar nuclei can be classified into an anterior and a posterior group. The anterior group comprises the central medial, paracentral and central lateral nuclei, and the posterior group comprises the centromedian (Cm) and parafascicular (Pf) nuclei (Figure 1B).

The MD thalamic nucleus can be divided into at least two parts based on cytoarchitectonics: a magnocellular division, located antero-medially, and a parvocellular division, located more laterally. Based largely on thalamocortical connectivity, Ray and Price (1993) further divided the magnocellular division into a paramedian subdivision proximal to the midline, and a fibrous subdivision. Separate from the parvocellular division, there is a poorly myelinated caudodorsal division. These different MD nucleus divisions preferentially and reciprocally connect with different prefrontal cortical (PFC) areas. The magnocellular division preferentially connects with orbitofrontal cortex (Brodmann areas (BA) 11, 13, 47/12), the caudodorsal division connects with medial PFC (BA 14, 24, 32), and the parvocellular division connects with lateral PFC (BA 9, 45, 46). Each MD thalamic division is well positioned to influence distinct PFC circuits.

The midline structure, the Re nucleus, is reciprocally connected with medial PFC and the hippocampal formation, subiculum and entorhinal cortex (rat: Vertes et al., 2006; Hoover and Vertes, 2012). Although it has been less studied, the Rh nucleus shows similar, possibly broader, connectivity. In addition, a number of Re/Rh neurons give collaterals to both PFC and medial temporal lobe (Cassel et al., 2013). It is important to note that the hippocampus directly projects to the PFC. In contrast, medial PFC directly projects to parahippocampal areas, but not to the hippocampus itself (Pandya et al., 1981; Vertes et al., 2007). Thus, information can only be indirectly routed from the medial PFC to the hippocampus, either via the Re/Rh or the parahippocampal areas (which themselves receive input from the Re/Rh). This anatomical connectivity suitably positions the midline thalamus to regulate communication between medial temporal and prefrontal areas.

The intralaminar thalamus has been classically viewed to non-specifically project to the cortex. However, more recent appraisals suggest that individual intralaminar nuclei each preferentially connect with particular regions of cortex (Van der Werf et al., 2002). In macaque monkeys, motor cortex and parietal cortex provide input to the central lateral nucleus, whereas the granular PFC (and medial limbic cortex) provides input to the central medial and paracentral nuclei (Künzle and Akert, 1977; Akert and Hartmann-Von Monakow, 1980). This anterior group of intralaminar thalamic nuclei also receives subcortical input from the cerebellum, brainstem and spinal cord (Jones, 2007). Evidence suggests at least the subcortical inputs from the cerebellum may predominantly synapse on intralaminar neurons that project to the striatum (see below; rat: Ichinohe et al., 2000). The anterior intralaminar nuclei project to frontal and parietal cortex. The central lateral and paracentral nuclei mainly project to the lateral cortical areas, whereas the central medial nucleus mainly projects to the medial and basal cortical areas. Projections to parietal cortex predominantly originate in the central lateral nucleus (cat: Macchi et al., 1984; Royce et al., 1989).

For the posterior group of intralaminar nuclei, the premotor cortex provides input to Pf and the motor cortex provides input to Cm (Künzle and Akert, 1977; Akert and Hartmann-Von Monakow, 1980; Chiba et al., 2001). The posterior intralaminar nuclei, predominantly Cm, receives a robust projection from the internal segment of the globus pallidus (Parent et al., 2001). Cm projects to motor cortex and Pf projects around rhinal sulcus and cingulate gyrus (cat: Macchi et al., 1984; Royce and Mourey, 1985). There is also a substantial projection from the intralaminar nuclei to the striatum, and it appears that more intralaminar neurons, especially in Cm, project to the striatum than the cerebral cortex.

The intralaminar nuclei project to most of the striatum, allowing modulation of striatal output (Smith et al., 2004). Generally speaking, cortical areas and intralaminar nuclei that are directly connected, also tend to project to overlapping parts of the striatum. Many cortical neurons projecting to the intralaminar nuclei have branched axons that project to the striatum as well (cat: Royce, 1983a; Paré and Smith, 1996). There is also a small number of Cm, Pf and central lateral neurons with axons that branch off to the striatum and cortex (cat: Royce, 1983b). Anterior intralaminar nuclei connections with the caudate and putamen mainly correspond with the location of cortico-striatal terminations from parietal and cingulate cortex. Considering the posterior intralaminar nuclei, there is a Cm connection bias towards the putamen and head of the caudate nucleus proximal to the internal capsule, where sensorimotor and premotor cortex projections terminate; and a Pf bias towards much of the caudate nucleus and anterior pole of the putamen, where PFC and parieto-temporal cortex projections terminate. That is, Cm projects to most of the sensorimotor area of the striatum (in dorsolateral caudate and putamen) and Pf projects to most of the cognitive and limbic areas of the striatum (in central caudate and putamen; Selemon and Goldman-Rakic, 1985; Sadikot et al., 1992). For more information on the thalamo-striatal pathway, see excellent recent reviews by Smith et al. (2004, 2011) and Minamimoto et al. (2014).

Summarizing the thalamo-cortical connectivity, the MD nucleus predominantly connects with the PFC, midline nuclei connect with medial PFC and medial temporal lobe, anterior intralaminar nuclei connect with frontal and parietal cortices, and posterior intralaminar nuclei connect with prefrontal and motor cortices (Figure 1C). The intralaminar and medial thalamic nuclei thus form important hubs in multiple frontal, parietal and medial temporal networks.

## Pulvinar as model for higher-order thalamus

The pulvinar is the largest thalamic nucleus and higher-order part of the visual thalamus. The pulvinar is extensively and reciprocally connected with much of the cerebral cortex, especially visual and oculomotor areas. Generally speaking, directly connected visual cortical areas are also indirectly connected via the pulvinar. This pattern of pulvinar connectivity with the cortex ideally positions the pulvinar to influence information transmission between cortical areas (Shipp, 2003; Sherman and Guillery, 2006; Saalmann and Kastner, 2011).

Recent studies have shown that lesions of the pulvinar can greatly reduce cortical excitability (Purushothaman et al., 2012) as well as produce profound behavioral deficits, including deficits in attention and sensory-guided actions (Snow et al., 2009; Wilke et al., 2010). Simultaneous recordings from the pulvinar and cortex have shown these areas synchronize their activity during attention tasks (Wróbel et al., 2007; Saalmann et al., 2012). Furthermore, evidence suggests that the pulvinar modulates the neural synchrony between cortical areas, to regulate information transmission across cortex according to behavioral demands (Saalmann et al., 2012). Because of common cellular properties across higher-order thalamic nuclei and thalamo-cortical connectivity patterns, a general role for the higher-order thalamus may be to modulate neural synchrony between groups of cortical neurons, to control cortical information transmission (Figure 1D). This idea will be explored in separate sections for the MD, midline and intralaminar thalamic nuclei.

## Mediodorsal thalamus physiology and function

Macaque monkey studies suggest a role for MD thalamic neurons in working memory processes. In macaques trained in delayed response tasks, MD neurons have been shown to modulate their spike rate during the cue, delay and/or response periods. Around half of neurons in the parvocellular division of the MD nucleus showed increased spike rate during the delay period of the task. Cells with delay period activity have been found in the magnocellular division as well, but the sample size is smaller (Fuster and Alexander, 1971, 1973; Watanabe and Funahashi, 2004). Such delay period activity has been proposed to play a key role in working memory processes. Many MD neurons also have shown direction selective activity reflecting the cue or upcoming response (Tanibuchi and Goldman-Rakic, 2003). The proportion of MD parvocellular neurons showing delay period activity and direction selectivity is similar to that in dorsolateral PFC (Takeda and Funahashi, 2002; Watanabe and Funahashi, 2004). Deactivation of the dorsolateral PFC reduced delay period activity in the parvocellular division of MD thalamus, and increased the number of MD thalamic neurons firing rhythmic bursts, typical of low vigilance states (Alexander and Fuster, 1973). This suggests that the PFC contributes to MD neuronal response characteristics.

In mice trained in a spatial working memory task (T maze delayed non-match to sample task), MD neuronal spiking synchronized with PFC local field potentials (LFPs) in the low beta frequency range (13–20 Hz), with MD spikes leading (Parnaudeau et al., 2013). During the task acquisition period, the beta frequency synchrony between the MD thalamus and PFC increased (measured as LFP-LFP coherence). Using a pharmacogenetic approach to hyperpolarize MD neurons, the reduced MD spiking activity corresponded to impaired performance of the mice in the delayed non-match to sample task (Parnaudeau et al., 2013). The reduced MD activity perturbed synchrony between MD and PFC. Importantly, the degree of MD-PFC synchronization correlated with task performance. Taken together, these results suggest that MD neurons influence PFC dynamics as well as working memory.

The PFC is vital for working memory as well as other executive brain functions, such as response inhibition, selective attention, and mental set shifting, which allow you to flexibly adapt your behavior according to current goals and context (Miller and Cohen, 2001; Diamond, 2013). Because different divisions of the MD nucleus connect with distinct regions of PFC (Ray and Price, 1993), which make differential contributions to executive processing, the MD nucleus may be involved in a variety of executive functions, not only working memory processes.

Because of the extensive reciprocal connections between the MD thalamus and PFC, one might expect that manipulating the MD thalamus will have an influence on the PFC, and vice versa. The above results are consistent with thalamo-cortical interactions playing an important role in working memory processes and perhaps executive processes more generally. The question posed here is how does the thalamus influence the cortex? Evidence suggests that directly connected PFC areas are also indirectly connected via the MD nucleus (Ray and Price, 1993). This connectivity pattern allows the MD thalamus to influence information transmission between PFC areas. Because this connectivity pattern is similar to that between the pulvinar and visual cortical areas, it is possible that the mechanism of MD influence on the cortex may also be similar to that of pulvinar influence on the cortex. That is, it is proposed that the MD thalamus normally modulates the degree of synchrony between different groups of PFC neurons, to regulate cortical information transmission. In this case, one would expect increased synchrony between the MD thalamus and PFC during working memory tasks, consistent with the Parnaudeau et al. (2013) study.

*A critical test of the proposal* would be to simultaneously record from the MD thalamus and two PFC areas during a working memory task. The prediction would be MD selectively synchronizing PFC neurons representing task relevant information (Figure 1D, top). *A second key test* would be deactivating MD thalamus to measure effects on synchrony and information transmission between PFC areas. The prediction would be abnormal cortical synchronization patterns and perturbed information transmission (Figure 1D, bottom). Interestingly, cortical synchronization patterns are altered in schizophrenia (Uhlhaas and Singer, 2010) and there is evidence of changes in MD thalamus as well (Andreasen, 1997; Popken et al., 2000; Alelú-Paz and Giménez-Amaya, 2008). One hypothesis consistent with these findings is that MD dysfunction in disorders such as schizophrenia may give rise to the observed changes in cortical synchronization patterns, which perturb information transmission and give rise to schizophrenic signs.

## Midline nuclei physiology and function

Evidence from rat studies suggests a role for the midline thalamus in memory processes. Increased neural activity in Re/Rh, gauged by c-Fos expression, has been shown 25 days after learning the Morris water maze (but not after 5 days). When Re/Rh was lesioned, there was normal acquisition of the water maze task, but impaired memory retrieval after 25 days (Loureiro et al., 2012). This suggests that Re/Rh contributes to memory consolidation. Re/Rh may further contribute to recognition memory, because Re/Rh lesions interfered with performance in a delayed non-match to sample task (Hembrook et al., 2012). The Re nucleus has also been implicated in fear memory, and it is has been proposed that Re regulates the generalization of memory attributes, to facilitate responses to novel situations that share similar features with past experiences (Xu and Sudhof, 2013). The role of the midline thalamic structures may not be limited to memory-related functions. Lesioning Re/Rh also has been reported to affect strategy shifting (Dolleman-Van Der Weel et al., 2009; Cholvin et al., 2013).

There have been relatively few electrophysiological recordings from the midline thalamic nuclei. In rats, systemic ketamine dosing (an NMDA receptor antagonist, here used to mimic schizophrenia symptoms) that slowed movements, but did not produce unconsciousness, increased the spike rate of Re neurons, the power of delta (1–4 Hz) oscillations in the Re nucleus, and the modulation of Re spiking activity at delta frequencies (locally applied ketamine induced a similar electrophysiological effect; Zhang et al., 2012). It has also been reported that the spike rate of Re neurons increased during theta (4–8 Hz) oscillatory activity induced by tail pinch (Morales et al., 2007). This suggests state-dependent modulation of both spike rate and spike timing in the Re nucleus.

The Re nucleus can synchronize with the hippocampus and induce hippocampal oscillatory patterns (Zhang et al., 2012). Dolleman-Van Der Weel et al. (1997) showed that stimulation of the Re nucleus caused subthreshold depolarization of pyramidal cells in hippocampus (CA1) and a suprathreshold excitation of inhibitory cells. Increasing Re output (with either neuroligin-2 knockdown or electrical stimulation) not only increased CA1 activity, but also increased medial PFC activity (measured using c-Fos expression: Xu and Sudhof, 2013; or evoked-potentials: Di Prisco and Vertes, 2006). Conversely, reducing Re output (tetanus toxin activation) reduced CA1 and anterior cingulate cortical activity (Xu and Sudhof, 2013). It has been shown that PFC neurons can synchronize their spiking to the hippocampal theta rhythm, with hippocampal activity leading PFC (Siapas et al., 2005). This prefrontal-hippocampal synchrony may be important for effective information transfer and spike timing-dependent plasticity. Because the Re nucleus has been shown to influence activity in both PFC and the hippocampus, as well as modulate oscillatory patterns in the hippocampus, it is possible that the Re modulates synchrony between the PFC and medial temporal lobe to regulate information transmission and storage.

## Anterior intralaminar nuclei physiology and function

It has been proposed that the anterior intralaminar nuclei are part of an oculomotor thalamus (Schlag, 2009). At least three types of anterior intralaminar neurons can be differentiated during spontaneous eye movements: burst neurons that increase firing around saccades; pause neurons that stop firing around saccades, a number with post-pause rebound activity; and eye-position neurons, whose activity reflects orientation of the eye in the orbit (Schlag-Rey and Schlag, 1984). In a delayed saccade task, anterior intralaminar neurons responded to the visual cue, delay period and/or saccade (Wyder et al., 2003). Most neurons showed motor-related or both visual and motor-related responses. These neurons showed directional tuning, even during delay activity. Only a small number of sampled neurons showed solely visual-related responses. The latency of responses to the visual cue was usually 60–100 ms, and neurons showing pre-saccadic activity as well as neurons showing post-saccadic activity were common. The timing of these saccade-related activities suggests that the anterior intralaminar thalamus may be able to contribute to saccade generation as well as movement monitoring, possibly corollary discharge processing.

Behavioral context influences the activity of anterior intralaminar neurons. Central thalamic neurons, including central lateral and paracentral neurons, have shown increased spiking activity during the delay period in visually-guided and memory-guided delayed saccade tasks (Wyder et al., 2003, 2004). This delay period activity was modulated based on whether the cue signaled a distractor or the saccade goal in the response field. Error trials influenced delay period activity as well (Wyder et al., 2004; Schiff et al., 2013). In a human positron emission tomography study, increased attentiveness during visual and somatosensory stimulus detection tasks activated the intralaminar nuclei, likely the central lateral and Cm nuclei (Kinomura et al., 1996). Oscillatory activity in the central thalamus also depends on context. Increased gamma frequency (30–100 Hz) power of LFPs, and reduced power at lower frequencies (10–20 Hz), has been reported during the delay period in a variable foreperiod, reaction time task (Schiff et al., 2013). The limited evidence available is consistent with the anterior intralaminar nuclei contributing to attention-related processes.

A classical finding is that electrically stimulating (e.g., at 6–14 Hz) the intralaminar, and a number of other, thalamic nuclei induces the cortical augmenting response, that is, increasing amplitude of cortical field potentials, with activity synchronizing over large cortical regions and post-augmenting oscillatory activity (Morison and Dempsey, 1943; Castro-Alamancos and Connors, 1996b; Steriade et al., 1998). The magnitude of the augmenting response depends on behavioral state: increasing vigilance reduces the augmenting response (Castro-Alamancos and Connors, 1996a). This may mean that the widespread cortical synchronization that disrupts normal information transmission, for instance, during anesthesia and sleep, gives way to more spatially precise synchronization during waking activities. It has been shown that anterior intralaminar thalamic stimulation produces robust evoked potentials in medial frontal and parietal cortex (rat LFP: Kung and Shyu, 2002; human EEG: Schiff et al., 2007) and optogenetic activation of thalamo-cortical axons modulates responses of neurons in cortical layer 1 and layer 2/3 (Cruikshank et al., 2012). This suggests that, under normal conditions, the anterior intralaminar nuclei can influence the excitability of frontal and parietal cortical neurons and synchronize these cortical neurons with spatial precision (not just expansive augmenting responses).

The intralaminar nuclei play an important role in arousal regulation. Central thalamic damage is associated with disorders of consciousness, including acute coma after bilateral lesions and hemispatial neglect after unilateral lesions (Schiff, 2008). In minimally conscious patients, electrical stimulation of the central thalamus can improve behavioral responsiveness (Schiff et al., 2007). Moreover, particular parts of the cortex appear to be important contributors to conscious awareness, including certain areas of parietal (e.g., posterior cingulate), temporo-parietal and frontal cortex (Hudetz, 2012), to which the intralaminar thalamic nuclei are extensively connected (Van der Werf et al., 2002). However, it is unclear how the intralaminar thalamus contributes to mechanisms of consciousness. A neural correlate of consciousness is large-scale integration of processing across multiple cortical areas (Alkire et al., 2008). This integration appears to rely at least in part on neural synchronization between distributed groups of cortical neurons. One hypothesis that ties together the above findings is that the intralaminar thalamus may precisely synchronize ensembles of cortical neurons in multiple circuits, including those related to orienting, attention and memory processes (processes which have been shown to influence conscious awareness). As a secondary effect, this thalamic-mediated cortical synchrony may give rise to large-scale integration of information, influencing the level of arousal and consciousness.

## Posterior intralaminar nuclei physiology and function

In macaque monkey studies, most Cm/Pf neurons show multimodal sensory activity, responding to auditory, visual and/or somatosensory stimuli. Cm/Pf neurons can be categorized into two types based on response latency: short- and long-latency facilitation neurons, with mean latency less than 100 ms (as short as 30 ms or less to auditory clicks) and greater than 200 ms respectively (Matsumoto et al., 2001; Minamimoto and Kimura, 2002). The short-latency neurons have been found predominantly in Pf and the long-latency neurons predominantly in Cm. Cm/Pf neurons generated brief, phasic responses to a stimulus, and a number of long-latency neurons generated two or three repeat phasic responses. Cm/Pf neurons generally showed greater responses to unexpected sensory stimuli, and habituated to repeated stimulus presentations. Cm/Pf neurons responded to the sensory stimuli whether or not they were associated with reward, unlike (tonically active) striatal neurons recorded under similar conditions, which only responded to stimuli linked to reward. Inactivating Cm/Pf greatly reduced the striatal response to the reward-linked stimuli; the effect on the cortex is not known (Matsumoto et al., 2001; Minamimoto and Kimura, 2002). Considering that attention-demanding tasks modulate Cm activity (Kinomura et al., 1996; Minamimoto and Kimura, 2002), these results suggest that the posterior intralaminar thalamus provides information about behaviorally relevant sensory events to the striatum, and possibly the cortical targets of intralaminar neurons as well. The posterior intralaminar nuclei may thus influence cortical processing through the thalamo-striatal input to cortico-striatal-thalamo-cortical pathways or, more directly, through the Cm/Pf input to the cortex.

Human subjects after thalamic stroke affecting the Cm/Pf (as well as ventral MD thalamus) have been reported to perform poorly on the Wisconsin Card Sorting Test. It was argued that the intralaminar thalamic lesion impaired shifting of cognitive sets (Liebermann et al., 2013). In macaques performing a go-nogo task with the go or nogo instruction associated with either a large or small reward, long-latency neurons in Cm showed greater activity during small reward trials than large reward trials (Minamimoto et al., 2005). The Cm response preceded movement execution. This suggests that the Cm helped counter bias (bias, in this case, toward the high reward action) when responding to external demands, thereby contributing to flexible shifts of rule-guided behavior. This interpretation is supported by the behavioral effects of electrical stimulation of Cm during the go-nogo task, that is, slowed responses for high reward actions (Minamimoto et al., 2005). Both the Wisconsin Card Sorting Test and the go-nogo task require cognitive flexibility and response inhibition. Different groups of neurons will need to be activated based on the current behavioral rule. Dynamically shifting between different rule-guided behaviors may involve synchronizing different task-relevant groups of frontal cortical neurons (Buschman et al., 2012). Synchrony between different cortical areas has been reported during the Wisconsin Card Sorting Test (González-Hernández et al., 2002), and Cm/Pf stimulation has been shown to synchronize cortical activity (Starzl and Magoun, 1951). This opens the possibility of Cm/Pf, in concert with other higher-order thalamic nuclei like MD, modulating cortical synchrony based on the current relevant course of action.

## Conclusion

Overall, the growing evidence supports important and specific roles for the intralaminar and medial nuclei, and higher-order thalamus more generally, in the control of cortical information transmission and cognitive processing. A critical mechanism may involve higher-order thalamus adjusting cortical synchrony and oscillatory patterns and thereby the efficacy of information transmission. However, further studies are needed to establish a causal role for the higher-order thalamus in regulating the synchrony between cortical neurons and consequently cognitive processing, particularly simultaneous neural recordings from thalamic and cortical areas of behaving primates as well as (pharmacological, electrical stimulation or optogenetic) manipulation of thalamo-cortical networks.

## Conflict of interest statement

The author declares that the research was conducted in the absence of any commercial or financial relationships that could be construed as a potential conflict of interest.
